# Genome-wide QTL analysis of meat quality-related traits in a large F_2_ intercross between Landrace and Korean native pigs

**DOI:** 10.1186/s12711-014-0080-6

**Published:** 2015-02-22

**Authors:** In-Cheol Cho, Chae-Kyoung Yoo, Jae-Bong Lee, Eun-Ji Jung, Sang-Hyun Han, Sung-Soo Lee, Moon-Suck Ko, Hyun-Tae Lim, Hee-Bok Park

**Affiliations:** Subtropical Animal Experiment Station, National Institute of Animal Science, Rural Development Administration, Jeju, 690-150 Korea; Department of Animal Science, College of Agriculture and Life Sciences, Gyeongsang National University, Jinju, 660-701 Korea; Institute of Agriculture and Life Science, Gyeongsang National University, Jinju, 660-701 Korea; Department of Animal Science and Biotechnology (BK21 plus program), College of Agriculture and Life Sciences, Chungnam National University, Daejeon, 305-764 Korea

## Abstract

**Background:**

We conducted a genome-wide linkage analysis to identify quantitative trait loci (QTL) that influence meat quality-related traits in a large F_2_ intercross between Landrace and Korean native pigs. Thirteen meat quality-related traits of the *m. longissimus lumborum et thoracis* were measured in more than 830 F_2_ progeny. All these animals were genotyped with 173 microsatellite markers located throughout the pig genome, and the GridQTL program based on the least squares regression model was used to perform the QTL analysis.

**Results:**

We identified 23 genome-wide significant QTL in eight chromosome regions (SSC1, 2, 6, 7, 9, 12, 13, and 16) (SSC for *Sus Scrofa*) and detected 51 suggestive QTL in the 17 chromosome regions. QTL that affect 10 meat quality traits were detected on SSC12 and were highly significant at the genome-wide level. In particular, the QTL with the largest effect affected crude fat percentage and explained 22.5% of the phenotypic variance (*F*-ratio = 278.0 under the additive model, nominal *P* = 5.5 × 10^−55^). Interestingly, the QTL on SSC12 that influenced meat quality traits showed an obvious trend for co-localization.

**Conclusions:**

Our results confirm several previously reported QTL. In addition, we identified novel QTL for meat quality traits, which together with the associated positional candidate genes improve the knowledge on the genetic structure that underlies genetic variation for meat quality traits in pigs.

**Electronic supplementary material:**

The online version of this article (doi:10.1186/s12711-014-0080-6) contains supplementary material, which is available to authorized users.

## Background

There are two types of Korean native pigs i.e. (1) native pigs raised on the Korean Peninsula and (2) the Jeju native pigs raised on the Jeju island. The Jeju native pig has unique genetic properties that differ from those of the Korea Peninsula pig because it is raised on an island that has been isolated for more than 1000 years (hereafter, the Jeju native pig is referred to as KNP). The coat color of KNP is black and its feed efficiency and growth rate are low, as for most native breeds. However, it has excellent meat quality characteristics such as a solid fat structure, white colored fat, rich meat juice, red meat color, and good marbling [1]. Nevertheless, studies on the genetic factors that influence the meat quality of KNP are still scarce.

To make pig quantitative trait locus (QTL) data publically available, a pig QTL database (pigQTLdb) was launched in 2004 (http://www.animalgenome.org/cgi-bin/QTLdb/SS/index). Since then, over 9800 QTL have been included into the pigQTLdb, for more than 650 different traits. These traits are classified into five categories (i.e., meat and carcass quality, reproduction, health, external appearance, and production). Among these five categories, meat and carcass quality are associated with the most abundant number of QTL (i.e., about 5400).

Although many studies that identified QTL for meat quality traits have been conducted, only a few causal mutations for meat quality-related traits have been identified. For example, the *RYR1* (*ryanodine receptor 1*, [2]) and *PRKAG3* (*protein kinase, AMP-activated, γ3 non-catalytic subunit*, [3]) genes are known to affect meat quality. *RYR1* on SSC6 (*Sus scrofa* chromosome 6) is involved in the control of intracellular calcium release that initiates contraction upon interaction with the voltage-dependent dihydropyridine receptor. Fujii *et al.* [2] identified a mutation in porcine *RYR1* (c.1843C>T) that is associated with higher lean meat percentage in skeletal muscle. This point mutation not only causes malignant hyperthermia but also a health problem known as porcine stress syndrome (PSS) and the development of pale, soft, exudative (PSE) meat that leads to major economic losses in the pork industry. The identification of this mutation provided a biochemical and physiological explanation for the origin of PSS and the occurrence of PSE meat in pigs. The *PRKAG3* gene on SSC15 encodes a muscle-specific isoform of the regulatory γ subunit of adenosine monophosphate-activated protein kinase (AMPK). A non-conserved substitution (p.Arg200Gln) in the *PRKAG3* gene was initially characterized in Hampshire pigs [3], and is thought to be a dominant negative mutation that inhibits glycogen degradation. In addition, it is well known that carriers of the *199V-200Q* haplotype show decreased protein content, ultimate pH, water holding capacity and processing yield, increased reflectance values, and a higher degree of protein denaturation compared with non-carriers [3-5].

In this study, we identified previously reported and novel QTL (i.e., that have not been reported previously in the literature) and associated positional candidate genes that may influence the meat properties of loin muscle (i.e., *m. longissimus lumborum et thoracis*) by using a large F_2_ intercross between Landrace and KNP pigs.

## Methods

### Animals and genotype analysis

A three-generation resource pedigree was generated and managed as described by Cho *et al.* [6]. Briefly, 19 purebred KNP were crossed with 17 purebred Landrace pigs. A total of 91 F_1_ progeny and 1105 F_2_ progeny (568 males and 537 females) from 79 full-sib families were produced. None of the F_2_ males were castrated. The Animal Care Committee at the Jeju National University approved all experimental procedures.

A total of 173 informative microsatellite (MS) markers that cover the autosomes and the X chromosome were amplified by PCR (polymerase chain reaction) for 1232 pigs, as described by Cho *et al.* [6]. The map orders and genetic distances were determined using the build option in the CRIMAP software version 2.4 [7]. The total map length was 2348.8 cM. The sex-average autosomal linkage map was used for further QTL analysis, except for the X chromosome.

### Phenotype analysis

All F_2_ pigs were slaughtered in the same commercial slaughterhouse. Before slaughter, pigs were fasted overnight during at least 16 hours, but with free access to water. The average age at slaughter was 199 days. The carcass samples were refrigerated 24 hours after pre-cooling.

After chilling for 24 hours, the loin muscle was boned and used to measure meat quality traits. Loin muscle area was recorded in two different ways i.e. (1) the *m. longissimus lumborum et thoracis* (MLLT) area between the 4th and 5th ribs (eye muscle area, EMA) and (2) the EMA including the *spinalis dorsi* muscle area between the 4th and 5th ribs (ALLEMA). We measured marbling score (MARB) of EMA subjectively according to the rules of the Korea Institute for Animal Products Quality Evaluation (KAPE), i.e., 1 meaning no marbling to 5 meaning over-abundant marbling. Crude fat (CFAT) in each collected EMA sample was extracted using chloroform–methanol (2:1, v/v) according to a standard procedure [8]. As described in Hwang *et al.* [9], CIE-value meat color (CIE-L, CIE-a, CIE-b), chroma (CHROMA), hue (HUE), shear force (SHEAR), moisture percentage (MOIST), and drip loss (DRIPL) were measured. Cooking loss (COOKL) was calculated as the weight of the cooked MLLT sample divided by the weight of the uncooked sample multiplied by 100.

### Statistical and QTL analyses

Before QTL analysis, descriptive statistics including phenotypic correlations were calculated and normal distribution of phenotype data was verified. Putative outliers were omitted based on the ascertainment of normality using the Ryan-Joiner (RJ) evaluation implemented in the MINITAB program (Minitab inc., USA). The RJ score ≥ 0.99 was used for the ascertainment of normality. Phenotypic values were transformed by natural logarithm when necessary. The following univariate animal model was fitted to the phenotype data to estimate heritabilities for each trait of interest:

$$ {\mathrm{Y}}_{\mathrm{i}\mathrm{jkl}}=\upmu +{\mathrm{s}}_{\mathrm{i}}+{\mathrm{p}}_{\mathrm{j}}+{\mathrm{b}}_{\mathrm{k}}\left(\mathrm{C}\mathrm{W}\right)+{\mathrm{a}}_{\mathrm{l}}+{\mathrm{e}}_{\mathrm{i}\mathrm{jkl}}, $$

where s_i_ is the fixed effect of the i^th^ sex; p_j_ is the fixed effect of the j^th^ parity; CW is the carcass weight of the k^th^ pig as a covariate; b_k_ is a fixed regression coefficient; a_l_ is the random additive polygenic effect of the l^th^ animal; and e_ijkl_ is the random residual effect. The mean and variance of the random residual effect of individuals were assumed to be: **e** ~ *N*(0, **I***σ*_*e*_^2^), where **I** is the identity matrix and *σ*_*e*_^2^ is the residual variance. The mean and variance of random additive polygenic effects can be defined as: **a** ~ *N*(0, **A***σ*_*a*_^2^), where **A** is the additive genetic relationship matrix computed from the F_2_ intercross pedigree in this study and *σ*_*a*_^2^ is the additive polygenic variance. The covariance between **a** and **e** was assumed to equal 0.

The following bivariate animal model was used to estimate genetic correlations among the 13 traits:

$$ \left[\begin{array}{c}\hfill {\mathbf{y}}_2\hfill \\ {}\hfill {\mathbf{y}}_2\hfill \end{array}\right]=\left[\begin{array}{cc}\hfill {\mathbf{X}}_1\hfill & \hfill 0\hfill \\ {}\hfill 0\hfill & \hfill {\mathbf{X}}_2\hfill \end{array}\right]\left[\begin{array}{c}\hfill {\mathbf{b}}_1\hfill \\ {}\hfill {\mathbf{b}}_2\hfill \end{array}\right]+\left[\begin{array}{cc}\hfill {\mathbf{Z}}_1\hfill & \hfill 0\hfill \\ {}\hfill 0\hfill & \hfill {\mathbf{Z}}_2\hfill \end{array}\right]\left[\begin{array}{c}\hfill {\mathbf{a}}_1\hfill \\ {}\hfill {\mathbf{a}}_2\hfill \end{array}\right]+\left[\begin{array}{c}\hfill {\mathbf{e}}_1\hfill \\ {}\hfill {\mathbf{e}}_2\hfill \end{array}\right], $$

where **y**_**1**_ and **y**_**2**_ are vectors of phenotypic measurements for the traits under consideration; **b**_**1**_ and **b**_**2**_ are vectors of fixed effects for the traits under consideration; **a**_**1**_ and **a**_**2**_ are vectors of the random additive polygenic effects for the traits under consideration; **X**_**1**_ and **X**_**2**_ are the incidence matrices relating records of the traits to fixed effects; **Z**_**1**_ and **Z**_**2**_ are the incidence matrices relating observations with random additive polygenic effects; **e**_**1**_ and **e**_**2**_ are vectors of random residuals. The expectation and variance of the bivariate animal model were:

$$ \mathrm{E}\left[\begin{array}{c}\hfill {\mathbf{y}}_2\hfill \\ {}\hfill {\mathbf{y}}_2\hfill \end{array}\right]=\left[\begin{array}{cc}\hfill {\mathbf{X}}_1\hfill & \hfill 0\hfill \\ {}\hfill 0\hfill & \hfill {\mathbf{X}}_2\hfill \end{array}\right]\left[\begin{array}{c}\hfill {\mathbf{b}}_1\hfill \\ {}\hfill {\mathbf{b}}_2\hfill \end{array}\right] $$

and

$$ \mathrm{V}\mathrm{a}\mathrm{r}\left[\begin{array}{c}\hfill {\mathbf{a}}_1\hfill \\ {}\hfill {\mathbf{a}}_2\hfill \\ {}\hfill {\mathbf{e}}_1\hfill \\ {}\hfill {\mathbf{e}}_2\hfill \end{array}\right]=\left[\begin{array}{llll}\mathbf{A}{\upsigma^2}_{\mathrm{a}1}\hfill & \mathbf{A}{\upsigma^2}_{\mathrm{a}12}\hfill & 0\hfill & 0\hfill \\ {}\mathbf{A}{\upsigma^2}_{\mathrm{a}21}\hfill & \mathbf{A}{\upsigma^2}_{\mathrm{a}2}\hfill & 0\hfill & 0\hfill \\ {}0\hfill & 0\hfill & \mathbf{I}{\upsigma^2}_{\mathrm{e}1}\hfill & \mathbf{I}{\upsigma^2}_{\mathrm{e}12}\hfill \\ {}0\hfill & 0\hfill & \mathbf{I}{\upsigma^2}_{\mathrm{e}21}\hfill & \mathbf{I}{\upsigma^2}_{\mathrm{e}2}\hfill \end{array}\right], $$

where **A** is the additive genetic relationship matrix; *σ*_a1_^2^, *σ*_a12_^2^, *σ*_a21_^2^, *σ*_a2_^2^ are additive polygenic (co)variances for the traits under consideration; *σ*_e1_^2^, *σ*_e12_^2^, *σ*_e21_^2^, *σ*_e2_^2^ are residual (co)variances for the traits; **I** is the identity matrix. **a** and **e** were assumed to be normally distributed with mean and (co)variances equal to 0 as mentioned above. All genetic parameters were computed by the Qxpak program with the REML (restricted maximum likelihood) option [10]. Phenotypic correlation coefficients were obtained by the MINITAB program (Minitab inc., USA).

The QTL analysis for each trait was performed using the web-based program GridQTL (http://www.gridqtl.org.uk). The interval mapping model based on the least squares regression method [11] was used for QTL analysis, including the cofactors of sex, parity, and carcass weight and additive and dominance regression variables for the putative QTL. Identification of QTL was based on an *F*-ratio test statistic that was calculated from sums of squares explained by the additive and dominance regression coefficients for the QTL. The *F*-ratios were calculated at 1 cM intervals throughout the genome. At the QTL peak, we extracted the additive and dominance coefficients for each F_2_ progeny to evaluate the significance of each additive and dominance effect using the MINITAB program and selected the final model for the QTL analysis. Nominal *P* < 0.05 was used as the significant threshold. Both the additive and the dominance regression coefficients were included in the QTL model if the effect of the dominance regression coefficients was significant, regardless of the significance level of the additive coefficient. Only the additive regression coefficient was included in the QTL model if the effect of the dominance regression coefficients was not significant. Incorporating previously detected QTL into the QTL model is expected to decrease the residual variance and thereby increase the statistical power to detect QTL with minor effects. Hence, the additive and dominance regression indicator variables for the most significant single QTL in the initial analysis were included as covariates, and a new genome scan was performed using the updated model. This process was repeated until no additional QTL was identified.

Statistical significant thresholds of the test statistic (i.e., *F*-ratio) was evaluated in each consecutive step in the QTL analysis procedure by 1000 permutation of data [12]. Genome-wide thresholds for highly significant (*α* = 0.01) and significant linkage (*α* = 0.05) were applied. Suggestive linkage was applied using a 5% chromosome-wide threshold. Confidence intervals (CI) of the identified QTL were estimated by the bootstrap resampling analysis with 2000 iterations [13].

## Results and discussion

We measured 13 traits that are considered as important in determining meat quality and performed a genome-wide linkage analysis to map QTL for these traits. Table 1 shows the descriptive statistics for the measured traits. Estimates of the genetic parameters of the traits are listed in Tables 1 and 2. Estimated heritabilities ranged from 23.2% (MARB) to 80.9% (HUE). Genetic and phenotypic correlations were also estimated between the traits used for heritability estimation. The trait CFAT was strongly correlated with MOIST (phenotypic correlation *r*_pheno_ = −0.695; genetic correlation *r*_geno_ = −0.691) and CIE-a (*r*_pheno_ = 0.591; *r*_geno_ = 0.751). Overall, both the magnitude and the sign of the genetic and phenotypic correlations did not differ significantly between traits (Table 2). In the genome-wide linkage analysis, we found 23 significant QTL (Table 3) and 51 suggestive QTL [See Additional file 1: Table S1]. The QTL results are described in detail in the following.

**Table 1:** **Descriptive statistics and heritabilities of meat quality traits in the KNP × Landrace intercross population**

**Traits**	**Abbreviation**	**N**	**Mean**	**SD**	**Range**	***h*** ^**2**^ **(%)**
Eye muscle area (cm^2^)	EMA	1047	21.27	3.811	8–35	34.1
Eye muscle area with *spinalis dorsi* muscle (cm^2^)	ALLEMA	1047	36.74	5.981	14–58	36.6
Marbling score in eye muscle area (KAPE)	MARB	831	1.86	0.958	1–5	23.2
Crude fat content in eye muscle area (%)*	CFAT	962 (3)	3.04	1.876	0.85–13.49	39.8
Shear force (kg/cm^2^)	SHEAR	956 (9)	3.53	0.992	0.86–7.49	61.2
Moisture percentage (%)	MOIST	958 (7)	73.82	1.409	67.63–77.83	37.3
Cooking loss (%)	COOKL	951 (13)	35.88	3.689	23.56–47.9	37.7
Drip loss (%)*	DRIPL	959 (6)	2.12	1.662	0.14–11.21	79.3
Meat color lightness	CIE–L	964	48.86	4.37	36.41–64.3	41.7
Meat color red/green*	CIE–a	963 (1)	8.4	2.558	2.92–18.94	44.1
Meat color yellow/blue*	CIE–b	954 (2)	3.71	2.979	−0.72–23.84	46.2
Meat color chroma*	CHROMA	962 (37)	9.7	3.868	2.98–32.08	42.4
Meat color hue	HUE	921 (43)	19.98	7.009	4.08–40.34	80.9

N = number of animals, values in parentheses are the number of animals excluded based on ascertainment of normality; *data transformed using natural logarithm.

**Table 2:** **Genetic (above diagonal) and phenotypic (below diagonal) correlations between 13 meat quality-related traits in an F**_2_ **intercross between KNP and Landrace**

	**EMA**	**ALLEMA**	**MARB**	**CFAT**	**SHEAR**	**MOIST**	**COOKL**	**DRIPL**	**CIE-L**	**CIE-a**	**CIE-b**	**CHROMA**	**HUE**
**EMA**	1	0.779	−0.195	−0.372	0.229	0.207	−0.004	0.128	0.006	−0.259	−0.221	−0.209	0.100
**ALLEMA**	0.779^‡^	1	−0.067	−0.318	0.070	0.239	0.020	0.070	−0.003	−0.380	−0.155	−0.101	0.052
**MARB**	−0.185^‡^	−0.096^†^	1	0.582	−0.254	−0.570	−0.107	−0.201	−0.031	0.250	0.197	0.184	0.047
**CFAT**	−0.340^‡^	−0.231^‡^	0.585^‡^	1	0.055	−0.691	−0.050	−0.391	0.033	0.751	0.253	0.587	0.368
**SHEAR**	0.248^‡^	0.186^‡^	−0.245^‡^	−0.247^‡^	1	0.160	0.297	0.682	0.197	0.445	0.017	0.248	0.090
**MOIST**	0.155^‡^	0.097^†^	−0.587^‡^	−0.695^‡^	0.150^‡^	1	0.076	0.160	−0.067	−0.343	−0.305	−0.318	−0.313
**COOKL**	^1^ N.E.	N.E.	−0.111^†^	N.E.	0.281^‡^	0.077*	1	0.296	0.455	0.184	0.202	0.267	0.421
**DRIPL**	0.127^‡^	N.E.	−0.178^‡^	−0.189^‡^	0.180^‡^	0.129^‡^	0.290^‡^	1	0.499	−0.190	−0.160	0.136	0.257
**CIE-L**	N.E.	N.E.	N.E.	N.E.	0.110^†^	−0.113^‡^	0.443^‡^	0.495^‡^	1	0.127	0.211	0.496	0.650
**CIE-a**	−0.234^‡^	−0.103^†^	0.230^‡^	0.591^‡^	N.E.	−0.321^‡^	0.162^‡^	N.E.	0.092^†^	1	0.656	0.733	0.509
**CIE-b**	−0.200^‡^	−0.098^†^	0.200^‡^	0.471^‡^	−0.072*	−0.311^‡^	0.188^‡^	N.E.	0.192^‡^	0.804^‡^	1	0.179	0.230
**CHROMA**	N.E.	N.E.	0.188^‡^	0.376^‡^	N.E.	−0.313^‡^	0.259^‡^	0.164^‡^	0.454^‡^	0.578^‡^	0.530^‡^	1	0.900
**HUE**	N.E.	N.E.	0.080*	0.187^‡^	0.154^‡^	−0.219^‡^	0.247^‡^	0.371^‡^	0.762^‡^	0.242^‡^	0.071*	0.652^‡^	1

*P < 0.05, ^†^ *P* < 0.01, ^‡^ *P* < 0.001, ^1^NE: estimated with no significance.

**Table 3:** **Summary of significant QTL for meat quality traits**

**QTL**	**SSC**	**Traits**	**Position (cM)**	***F*** **-ratio** ^**a**^	**Inheritance mode** ^**b**^	**95% Confidence interval** ^**c**^		**Var %** ^**d**^	**Additive ± SE** ^**e**^	**Dominance ± SE** ^**f**^	**Covariate QTL**
						**cM**	**Marker**				
	1	MARB	77	14.6*	A	0–142	*SW1514-SW373*	1.7	−0.171 ± 0.045		Q9
Q1		CFAT	152	19.8**	A	0–164	*SW1514-SW1301*	2.0	−0.132 ± 0.030		
	2	EMA	6	13.8*	A	0–40	*SW2623-SW240*	1.3	−0.604 ± 0.163		Q3, Q7
Q2		ALLEMA	3	19.6**	A	0–11	*SW2623-SW256*	1.9	−1.123 ± 0.254		
Q3	6	EMA	80	24.0**	A	76–84	*SW492-SW122*	2.3	−0.763 ± 0.156		
Q4		ALLEMA	77	16.7**	A	35.5–86	*SW2406-S0059*	1.6	−0.954 ± 0.233		
Q5	7	CFAT	55	15.3**	A	27–103	*SW1354-SW2108*	1.6	0.097 ± 0.025		
Q6	9	CIE-b	145	14.0**	AD	142.5–145	*SW2093-SW749*	2.9	0.061 ± 0.019	0.160 ± 0.040	
Q7	12	EMA	104	58.3**	A	102–111	*S0106-SWR1021*	5.3	−1.123 ± 0.147		
Q8		ALLEMA	107	22.7**	A	96–115	*S0106-SWR1021*	2.1	−1.082 ± 0.227		
Q9		MARB	107	68.3**	A	98–113	*S0106-SWR1021*	7.6	0.367 ± 0.044		
Q10		CFAT	101	278.0**	A	99–110	*S0106-SWR1021*	22.5	0.350 ± 0.021		
Q11		SHEAR	110	48.6**	A	98–115	*S0106-SWR1021*	4.9	−0.321 ± 0.046		
Q13		DRIPL	108	30.7**	A	96–115	*S0106-SWR1021*	3.1	−0.193 ± 0.035		
Q12		MOIST	107	79.0**	A	98–112	*S0106-SWR1021*	7.7	−0.521 ± 0.059		
Q14		CIE-a	102	83.9**	AD	99–110	*S0106-SWR1021*	14.9	0.153 ± 0.012	0.041 ± 0.019	
Q15		CIE-b	108	93.8**	A	100–114	*S0106-SWR1021*	8.9	0.158 ± 0.016		
Q16		CHROMA	112	29.8**	A	85–115	*SW1962-SWR1021*	3.1	0.165 ± 0.030		
	13	EMA	26	15.9*	A	0–37	*SWR1941-SW864*	1.5	0.609 ± 0.153		Q3, Q7
		ALLEMA	25	13.9*	A	3–40	*SWR1941-S0283*	1.3	0.836 ± 0.224		Q2, Q4, Q8
		CFAT	30	14.4*	A	21–57	*SW1407-S0283*	1.5	−0.078 ± 0.021		Q1, Q5, Q10
	16	CFAT	51	8.6*	AD	37–62	*SW419-SW2517*	1.8	−0.048 ± 0.029	−0.165 ± 0.044	
		CIE-b	58	9.1*	AD	26.5–94	*SW1035-S0105*	1.9	−0.055 ± 0.019	−0.086 ± 0.029	Q6, Q15

^a^Test statistic and level of genome-wide significance (**1%, *5%) thresholds; ^b^A represents additive effect and AD represents additive and dominance effects; ^c^confidence intervals estimated by the bootstrap analysis method, Marker indicates the flanking markers for the QTL confidence intervals; ^d^Var % is the reduction in residual variance of the F_2_ population obtained by inclusion of a QTL at the given position; ^e^additive effect and standard error, a positive value means that the Jeju native pig allele has a positive effect on a trait, and a negative value indicates that the Landrace allele has a positive effect on a trait; ^f^dominance effect and standard error.

### Eye muscle area (EMA)

EMA is one of the major economic traits in pigs, since it is proportional to the muscle mass of the carcass. On SSC6, QTL that affected EMA and ALLEMA accounted for up to 2.3% of the phenotypic variance. In this QTL region, Yue *et al.* [14] identified *RYR1* as a positional candidate gene for EMA and also reported that it influenced growth and carcass quality. Moreover, Grindflek *et al.* [15] reported a muscling QTL in the region near *RYR1*. In the same region, Mohrmann *et al.* [16] reported a QTL for EMA between the 13th and 14th ribs. On SSC12, we identified a QTL that affected EMA with an *F*-ratio of 58.3 (nominal *P*-value = 5.1 × 10^−14^) and that accounted for 5.3% of the phenotypic variance. The QTL for ALLEMA co-localized with the QTL for EMA. This QTL explained 2.1% of the phenotypic variance and overlapped with a suggestive QTL reported by Ponsuksili *et al.* [17]. This region contains the *SRY-related HMG-box 15* (*SOX15*) gene, which plays a role in the regulation of skeletal muscle myogenesis [18]. Thus, we suggest that *SOX15* can be a potential candidate gene for EMA in pigs. Interestingly, all the significant QTL for EMA and ALLEMA showed an additive inheritance mode and all alleles at these QTL from the Landrace breed were associated with higher phenotypic values for these traits.

### Marbling and crude fat percentage

Intramuscular fat (IMF), which is called marbling (MARB) in meat, usually accumulates in the loose membrane of both the perimysium and epimysium connective tissues in muscle. More than 90% of the accumulated IMF is composed of neutral lipids. Crude fat (CFAT) percentage is the relative amount of neutral fat in muscle and, in general amounts to the same quantity as total IMF content. We found significant QTL for CFAT in five chromosomal regions (i.e., SSC1, 7, 12, 13, and 16).

On SSC1, QTL that affected MARB and CFAT were located in different regions and explained 1.7% and 2.0% of the phenotypic variance, respectively. The QTL identified for MARB in this study overlapped with previously reported QTL regions for the same trait [19-22]. Previously identified QTL for IMF overlapped with the QTL region for CFAT that we found in this study [23-25].

**Figure 1:**
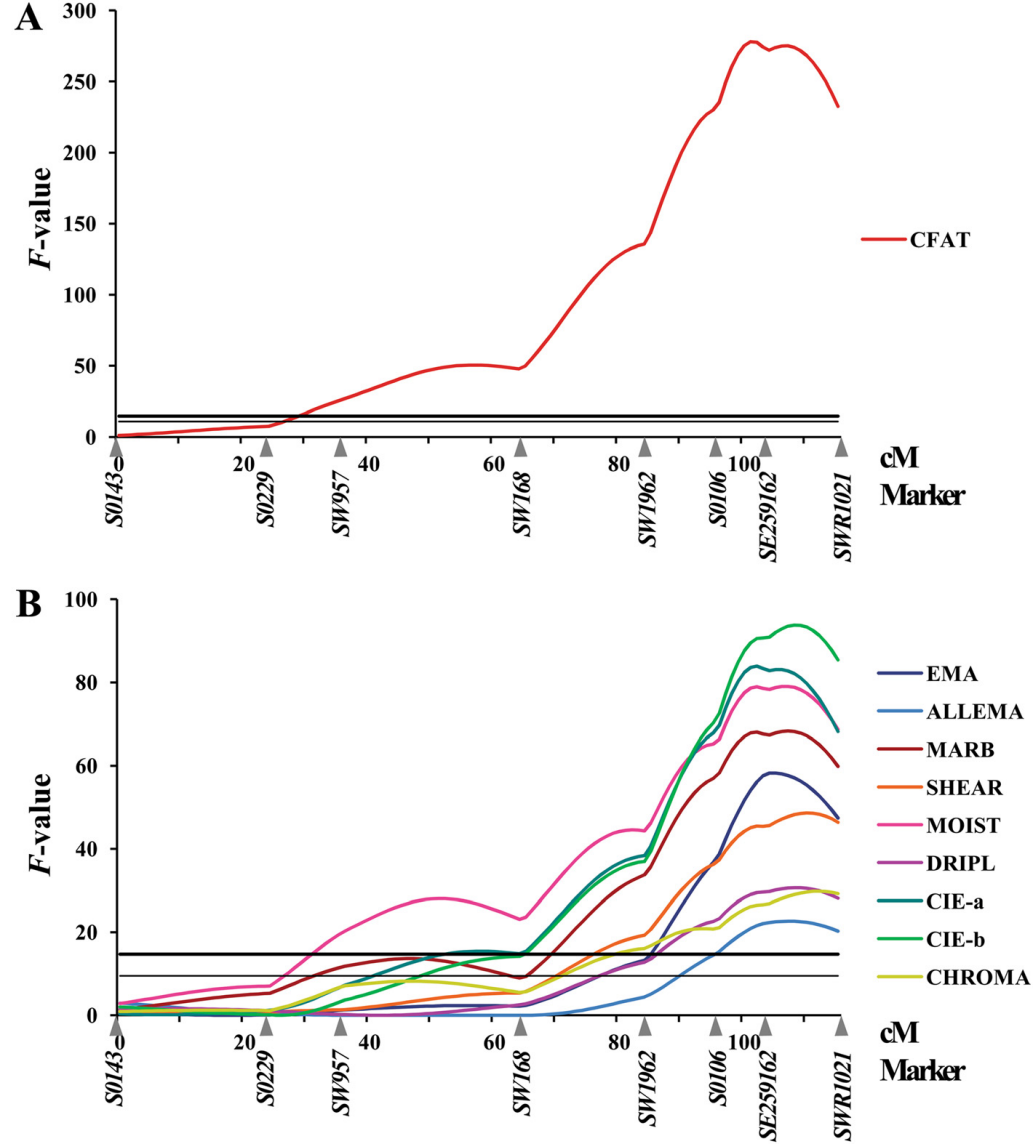
**QTL profiles for meat quality related traits on SSC12.** The y-axis represents the F-value testing the hypothesis of a single QTL on a given position on the chromosome. The marker map with genetic distance between microsatellite (MS) markers in Kosambi cM is given on the x-axis. The thick horizontal line indicates the 1% genome-wide significant threshold and thin horizontal line indicates the 5% chromosome-wide significant threshold. Trait abbreviations are in Table 1. **(A)** QTL profile for CFAT. **(B)** QTL profiles for EMA (eye muscle are), ALLEMA (EMA area and *spinalis dorsi* muscle), MARB (marbling score in EMA), SHEAR (shear force), MOIST (moisture percentage), DRIPL (drip loss), CIE-a (meat color red/green), CIE-b (meat color yellow/blue), and CHROMA (meat color chroma).

On SSC12, we identified a major additive QTL for CFAT (*F*-ratio = 278.0, nominal *P*-value = 5.5 × 10^−55^) (Figure 1A). This QTL was the most significant QTL detected in this study and explained 22.5% of the phenotypic variance. The allele of this QTL present in the KNP breed was found to be associated with higher phenotypic values of CFAT. A highly significant QTL for MARB was also identified in the same region, with an *F*-ratio of 68.3 (nominal *P*-value = 5.5 × 10^−16^) and accounting for 7.6% of the phenotypic variance. Previous studies reported that a cluster of genes on SSC12 that encode the myosin heavy chain (MYH) was associated with IMF [26,27]. However, the genetic map used in our study did not include *MYH* loci. Thus, further studies are necessary to investigate the effects of *MYH* loci on CFAT and MARB. An allele present in the KNP breed was associated with higher phenotypic values of MARB. Interestingly, this QTL region overlapped with those of EMA, ALLEMA, SHEAR, MOIST and DRIPL in this study. The same region also influenced backfat thickness between the 4th and 5th ribs [28].

### Shear force

Shear force (SHEAR), which is the force required to masticate meat in the mouth, is an indicator of meat tenderness. A well-known candidate gene is the *calpastatin* (*CAST*) gene on SSC2. According to Ciobanu *et al.* [29], calpastatin inhibits both μ- and m-calpains and controls the calpain system as well as post-slaughter softening of the carcass. In addition, it is very closely associated with SHEAR. However, we identified a novel significant QTL for SHEAR on SSC12 only. This QTL explained 4.9% of the phenotypic variance. The allele present in the KNP breed was associated with lower phenotypic values of SHEAR. After incorporating this QTL as a covariate in the multiple QTL model, we detected an additional novel QTL for SHEAR on SSC13 with suggestive significance [See Additional file 1: Table S1].

### Meat color

Meat color plays an important visual role when consumers choose fresh meat. In this study, meat color traits were recorded by an instrument that measures light wavelength. We identified genome-wide significant QTL in three chromosome regions (SSC9, 12, and 16). Redness (CIE-*a*) of meat color is determined by the molecule that is attached to the 6th binding site of the iron atom located at the center of the heme ring (porphyrin ring) that is the non-protein part of the meat color pigment (myoglobin) [30]. We identified only one genome-wide significant QTL for CIE-*a* at position 102 cM on SSC12. The QTL explained 14.9% of the phenotype variance. The KNP allele showed a positive effect on CIE-*a*. Yellowness of meat (CIE-*b*) is strongly influenced by the fat deposits in muscle. Generally, yellowness increases as the amount of fat deposited in the muscle increases. We identified novel significant QTL for CIE-*b* on SSC9, 12, and 16, which had not been previously reported. The KNP allele showed a positive effect for the two QTL on SSC9 and SSC12, but a negative effect for the QTL on SSC16. CHROMA i.e. pure color is affected by the mixing ratio of white light and is proportional to the absolute values of CIE-*a* and -*b* [31]. On SSC12, a QTL was identified in the same region as the QTL for CIE-*a* and -*b*. The KNP allele showed a positive effect as for the other meat color QTL on SSC12. In addition, we identified novel suggestive QTL on SSC3 (CHROMA), SSC10 (CIE-b), and SSC16 (CIE-a and CHROMA) after including previously detected QTL as covariates in the QTL model [See Additional file 1: Table S1].

### Moisture percentage, drip loss, and cooking loss

Water holding capacity WHC is defined by the ability of the meat to hold water during the application of a physical treatment and is closely related with meat texture and moisture percentage. We analyzed the QTL for moisture-related traits (MOIST, COOKL, and DRIPL) of the MLLT and identified significant QTL for MOIST (107 cM) and DRIPL (108 cM) on SSC12. These QTL regions overlapped with the QTL region for CFAT on SSC12 identified in this study. Previously, Edwards *et al*. [32] reported that CFAT and moisture percentage are closely correlated to each other in the MLLT. In agreement with this report, we found that QTL that affect MOIST and DRIPL co-localized with the QTL for CFAT. We also detected novel suggestive QTL for MOIST on SSC8 (3 cM) and SSC12 (41 cM) after applying the multiple QTL model [See Additional file 1: Table S1].

## Conclusions

In this study, we performed a genome-wide QTL analysis for meat quality-related traits using a large F_2_ intercross between Landrace and KNP pigs. The results not only validated some previously reported QTL but also detected novel chromosomal regions that were significantly associated with meat quality-related traits in pigs. Moreover, we showed that the QTL on SSC12 affected multiple meat quality traits (i.e., EMA, ALLEMA, SHEAR, MOIST, DRIPL, CIE-a, CIE-b, CHROMA, MARB and CFAT) (Figure 1). This co-localization of multiple QTL for meat quality-related traits suggests a genetic correlation among traits. In fact, moderate to high values of genetic correlation coefficient were observed between CFAT and the multiple meat quality traits except for SHEAR (Table 2). Mechanisms that explain genetic correlations can be either the presence of pleiotropic QTL or tightly linked QTL. However, in this study, the average marker density was one marker per 13.6 cM, which is too sparse to evaluate pleiotropy vs. linkage on a fine scale. Moreover, adding more markers would likely not have contributed in separating QTL since linkage disequilibrium is too extensive in an F_2_ intercross. The results presented here should be useful to investigate the genetic structure that underlies genetic variation of meat quality-related traits.

## Additional file

Additional file 1: Table S1. Summary of suggestive QTL for meat quality traits. The QTL locations, confidence intervals, test statistics (*F*-ratio), percentages of the variation explained (%), additive (a), dominance genotypic values (d) for QTL together with mode of inheritance, and covariate QTL information; all *F*-ratio values are suggestively significant at the 5% chromosome-wise level.
