# Supplementary material for: Genome-wide QTL analysis of meat quality-related traits in a large F2 intercross between Landrace and Korean native pigs
Source: Genet Sel Evol. 2015 Feb 22;47(1):7. doi: 10.1186/s12711-014-0080-6 (PMC4336478; doi:10.1186/s12711-014-0080-6)
Supplement: Additional file 1: Table S1. — Summary of suggestive QTL for meat quality traits. The QTL locations, confidence intervals, test statistics (F-ratio), percentages of the variation explained (%), additive (a), dominance genotypic values (d) for QTL together with mode of inheritance, and covariate QTL information; all F-ratio values are suggestively significant at the 5% chromosome-wise level. [file 12711_2014_80_MOESM1_ESM.docx]

**Supplementary table 1. Summary of suggestive QTLs for meat quality traits.**

| **SSC** | **Traits** | **Position**  **(cM)** | ***F*-ratio ^a^** | **Inheritance**  **mode ^b^** | **95% Confidence Interval ^c^** | | **Var**  **% ^d^** | **Additive**  **± SE ^e^** | **Dominance**  **± SE ^f^** | **Covariates**  **QTL** |
| --- | --- | --- | --- | --- | --- | --- | --- | --- | --- | --- |
|  |  |  |  |  | **cM** | **Marker** |  |  |  |  |
| 1 | EMA | 115 | 8.4 | A | 0-167 | *SW1514-SW1301* | 0.8 | -0.468 ± 0.162 |  | Q3, Q7 |
|  | MOIST | 2 | 8.4 | A | 0-159 | *SW1514-SW1301* | 0.9 | -0.181 ± 0.063 |  |  |
| 2 | SHEAR | 82 | 7.1 | A | 4-105 | *SW2623-SW1879* | 0.7 | 0.136 ± 0.051 |  | Q11 |
|  | DRIPL | 29 | 8.5 | A | 11-90 | *SW256-S0370* | 0.9 | -0.109 ± 0.037 |  | Q13 |
|  | CIE-L | 6 | 7.6 | A | 0-105 | *SW2623-SW1879* | 0.8 | -0.609 ± 0.222 |  |  |
| 3 | ALLEMA | 74 | 6.1 | AD | 12.5-135 | *APR22-SW2532* | 1.2 | -0.049 ± 0.239 | 1.337 ± 0.382 |  |
|  | SHEAR | 37 | 8.2 | A | 7-133.5 | *APR22-SW2532* | 0.9 | -0.152 ± 0.053 |  | Q11 |
|  | COOKL | 117 | 5.9 | AD | 21-132 | *SW2021-SW2532* | 1.2 | -0.141 ± 0.187 | 1.024 ± 0.305 |  |
|  | CIE-b | 151 | 11.3 | A | 0-151 | *APR22-SW2532* | 1.2 | -0.055 ± 0.016 |  |  |
|  | CHROMA | 142 | 7.2 | A | 0-151 | *APR22-SW2532* | 0.8 | -0.085 ± 0.032 |  | Q16 |
| 4 | MARB | 44 | 8.9 | A | 20-108 | *S0301-MP77* | 1.1 | 0.133 ± 0.044 |  |  |
|  | SHEAR | 13 | 10.1 | A | 0-126 | *SW489-MP77* | 1.1 | -0.155 ± 0.049 |  | Q11 |
|  | DRIPL | 44 | 7.9 | A | 0-126 | *SW489-MP77* | 0.8 | -0.096 ± 0.034 |  |  |
| 5 | ALLEMA | 0 | 11.4 | A | 0-116 | *SW413-SW1982* | 1.1 | -0.846 ± 0.250 |  | Q2, Q4, Q8 |
|  | MARB | 87 | 6 | AD | 22-150 | *SW1482-SW967* | 1.4 | -0.090 ± 0.046 | -0.178 ± 0.065 |  |
|  | CIE-b | 26 | 7.8 | A | 22-123 | *SW1482-SW1383* | 0.8 | -0.050 ± 0.018 |  | Q6, Q15 |
|  | CIE-L | 75 | 5.7 | AD | 7.5-122.5 | *SW413-SW1383* | 1.2 | -0.093 ± 0.224 | 1.153 ± 0.343 |  |
| 6 | MARB | 80 | 10.5 | A | 9-137 | *S0035-SW322* | 1.3 | 0.142 ± 0.044 |  | Q9 |
|  | CFAT | 47 | 11.3 | A | 42-137 | *SW1841-SW322* | 1.2 | 0.070 ± 0.021 |  | Q1, Q5, Q10 |
|  | SHEAR | 86 | 12.3 | A | 68-158 | *APR8-SW2419* | 1.3 | -0.154 ± 0.044 |  | Q11 |
| 7 | ALLEMA | 15 | 8.7 | A | 7-119 | *SW1873-SW2108* | 0.8 | 0.699 ± 0.237 |  | Q2, Q4, Q8 |
|  | MOIST | 54 | 11.8 | A | 50-126 | *207G8-4-SW2108* | 1.2 | -0.203 ± 0.059 |  | Q12 |
|  | CIE-a | 55 | 11.8 | A | 7-83 | *SW1873-SW147* | 1.2 | 0.047 ± 0.014 |  |  |
| 8 | ALLEMA | 77 | 10.8 | A | 15-158 | *S0353-KS188* | 1.0 | -0.750 ± 0.228 |  | Q2, Q4, Q8 |
|  | MOIST | 3 | 11.9 | A | 0-108 | *SW2410-S0225* | 1.2 | -0.218 ± 0.063 |  | Q12 |
| 9 | MOIST | 65 | 11.5 | A | 8-145 | *SY3-SW749* | 1.2 | -0.202 ± 0.060 |  | Q12 |
|  | CIE-a | 145 | 8.0 | AD | 16.5-145 | *SY3-SW749* | 1.7 | 0.034 ± 0.014 | 0.090 ± 0.030 | Q14 |
|  | CIE-L | 129 | 7.5 | A | 15-145 | *SY3-SW749* | 0.8 | 0.769 ± 0.282 |  |  |
| 10 | MARB | 122 | 7.5 | A | 27-135 | *SWR136-SW2067* | 0.9 | -0.190 ± 0.069 |  |  |
|  | SHEAR | 98 | 10.2 | A | 0-104 | *SW830-SW2067* | 1.1 | -0.144 ± 0.045 |  | Q11 |
|  | CIE-a | 92 | 11.1 | A | 0-128 | *SW830-SW2067* | 1.1 | -0.044 ± 0.013 |  |  |
|  | CIE-b | 92 | 7.7 | A | 1-135 | *SW830-SW2067* | 0.8 | -0.044 ± 0.016 |  | Q6, Q15 |
| 11 | ALLEMA | 77 | 6.1 | AD | 6-81 | *SW1632-SW1135* | 1.2 | -0.520 ± 0.271 | -1.313 ± 0.454 |  |
|  | CIE-b | 57 | 7.8 | A | 6-64 | *SW1632-SW1135* | 0.8 | -0.059 ± 0.021 |  |  |
| 12 | MOIST | 41 | 7.7 | A | 0-104 | *S0143-SE259162* | 0.8 | -0.191 ± 0.069 |  | Q12 |
| 13 | SHEAR | 27 | 10.6 | A | 6-69 | *SW1378-SW1386* | 1.1 | -0.143 ± 0.044 |  | Q11 |
|  | MOIST | 51 | 8.7 | A | 24-75 | *SW1407-SW1386* | 0.9 | 0.189 ± 0.064 |  | Q12 |
|  | CIE-a | 23 | 6.1 | AD | 7-99 | *SW1378-KS604* | 1.3 | -0.033 ± 0.012 | -0.041 ± 0.019 | Q14 |
|  | HUE | 50 | 8.2 | AD | 12-94 | *SW1378-SW38* | 1.8 | -1.030 ± 0.364 | -1.866 ± 0.642 |  |
| 14 | CIE-L | 4 | 8 | A | 0-107 | *SW857-SW2515* | 0.8 | -0.629 ± 0.222 |  |  |
|  | HUE | 0 | 7.8 | A | 0-101 | *SW857-SW2515* | 0.8 | -0.985 ± 0.353 |  |  |
| 15 | EMA | 125 | 6.3 | AD | 0-154.5 | *KS911-SWR2121* | 1.2 | -0.396 ± 0.176 | -0.786 ± 0.283 |  |
|  | ALLEMA | 110 | 8.3 | A | 6-152 | *KS911-SWR2121* | 0.8 | -0.648 ± 0.225 |  |  |
|  | SHEAR | 150 | 8.3 | A | 14-155 | *S0355-SWR2121* | 0.9 | -0.147 ± 0.051 |  |  |
| 16 | EMA | 57 | 6.4 | AD | 30-97 | *SW419-S0105* | 1.2 | 0.275 ± 0.186 | 0.931 ± 0.295 |  |
|  | COOKL | 36 | 7.1 | AD | 21.5-88 | *SW1035-S0105* | 1.5 | 0.053 ± 0.197 | -1.158 ± 0.307 |  |
|  | CIE-a | 56 | 7.7 | AD | 24-91 | *SW1035-S0105* | 1.6 | -0.044 ± 0.015 | -0.056 ± 0.023 | Q14 |
|  | CHROMA | 68 | 8.2 | A | 0.5-85 | *S0111-SW1897* | 0.9 | -0.086 ± 0.030 |  | Q16 |
| 18 | CIE-a | 44 | 6.7 | A | 0-44 | *SY4-SW1682* | 0.7 | 0.038 ± 0.014 |  |  |
| X | ALLEMA | 67 | 7.4 | A | 10-126 | *SW949-SJ017* | 1.5 | -0.900 ± 0.329 |  |  |
|  | MOIST | 100 | 8.7 | A | 6-126 | *SW949-SJ017* | 1.9 | -0.316 ± 0.107 |  |  |

^a^ Test statistic and level of significance genome-wide significance (^**^1%, ^*^5%) thresholds. ^b^ A represents additive effect; AD represents additive and dominance effects. ^c^ Confidence intervals estimated by the bootstrap analysis method; Marker means flanking markers for the QTL confidence intervals. ^d^ Var % is the reduction in residual variance of the F_2_ population obtained by inclusion of a QTL at the given position. **^e^** Additive effect and standard error. A positive value means the Jeju native pig allele has an increase effect on a trait, and a negative value indicates that the Landrace allele has an increase effect on a trait. ^f^ Dominance effect and standard error.
